# Global, Regional, and National Prevalence of Chronic Type 2 Diabetic Kidney Disease From 1990 to 2021: A Trend and Health Inequality Analyses Based on the Global Burden of Disease Study 2021

**DOI:** 10.1111/1753-0407.70098

**Published:** 2025-05-22

**Authors:** Yujun He, Xiaoyi Wang, Lu Li, Minhui Liu, Yachao Wu, Ru Chen, Jiujie He, Wei Mai, Xiaojun Li

**Affiliations:** ^1^ Department of Traditional Chinese Medicine Taizhou Hospital of Zhejiang Province Affiliated to Wenzhou Medical University Linhai, Taizhou Zhejiang People's Republic of China; ^2^ Guangxi Medical University Cancer Hospital Nanning Guangxi People's Republic of China

**Keywords:** chronic type 2 diabetic kidney disease, GBD, health inequality, prediction, trend

## Abstract

**Background:**

Diabetic kidney disease (DKD) is a prevalent and severe complication of diabetes that significantly impacts global health and quality of life. Most DKD is attributable to type 2 diabetes; therefore, chronic type 2 DKD warrants further examination.

**Objective:**

To deliver targeted assistance in alleviating the worldwide, regional, and national burden of chronic type 2 DKD, we executed a survey assessing the prevalence of chronic type 2 DKD utilizing the Global Burden of Disease, Injury, and Risk Factors (GBD) database.

**Methods:**

We examined the temporal trends of chronic type 2 DKD prevalence over the past 30 years using the 2021 GBD database, analyzed the trends by population, epidemiological change, and aging, and quantified cross‐country health inequalities. Additionally, we forecasted the trend during the subsequent two decades.

**Results:**

In 2021, there were over 107 million cases of chronic type 2 DKD globally, reflecting an 85.11% rise from 58 million cases in 1990. The age‐standardized rate (ASR) declined with an estimated annual percentage change of 0.17% per annum. Epidemiological change and population expansion are the primary factors influencing the alterations. The contributions of epidemiological change, population, and aging vary with alterations in the sociodemographic index (SDI). Significant health inequalities were observed across 204 countries and territories, with the slope index of inequality increasing over time. The forecast for the worldwide burden of chronic type 2 DKD from 2020 to 2040 suggests a significant rise in case numbers, while the alterations in ASR remain largely stable.

**Conclusions:**

These findings indicate the significant disease burden of chronic type 2 DKD, necessitating more targeted and effective interventions for its prevention and management.

## Graphical abstract

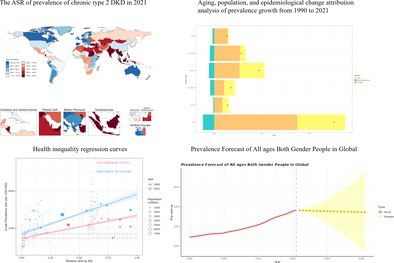

**Summary**

- Compared to 1990, the prevalence case number of chronic type 2 DKD in 2021 has significantly increased.
- Population growth and aging are the primary factors influencing the alterations of chronic type 2 DKD.
- Chronic type 2 DKD needs more targeted and effective interventions for its prevention and management.

## 1 Introduction

Diabetes is a growing global concern affecting around 9.3% of individuals aged 20 to 79 worldwide [1]. In 2019, over 463 million persons received a diabetes diagnosis. The worldwide prevalence of diabetes is anticipated to increase from 8.4% in 2017 to 9.9% by 2045. Diabetes is a chronic disorder generally classified into type 1 and type 2, with type 2 diabetes mellitus (T2DM) being the predominant form [1, 2], including 90% of diabetes cases [3, 4]. Diabetic kidney disease (DKD), as a common complication of diabetes, is the leading type of chronic kidney disease (CKD) and the principal cause of end‐stage kidney disease, accounting for more than 50% of all end‐stage kidney disease patients globally [5, 6]. The Global Burden of Disease (GBD) 2021 classifies CKD into five categories: CKD due to type 1 diabetes mellitus, CKD due to type 2 diabetes mellitus, CKD due to glomerulonephritis, CKD due to hypertension, and CKD due to other and unspecified causes. It is estimated that T2DM constitutes 30%–50% of the various etiologies of CKD [7]. In 2019, T2DM was the second predominant cause of CKD and CKD‐associated mortality globally, with 2.62 million cases of diabetes mellitus‐related CKD comorbidity reported worldwide [8]. The increasing prevalence of chronic type 2 DKD would markedly augment the disease burden due to its adverse impact on quality of life and the considerable expenses linked to its extended course [7, 9]. Therefore, greater emphasis should be placed on DKD resulting from T2DM in comparison to DKD resulting from type 1 diabetes.

The Global Disease, Injury, and Risk Factor Assessment model aims to elucidate epidemiological change by quantifying disease risk factors, characterizing diseases, and evaluating disease burden, while also providing insights and data to facilitate global health solutions [8]. The financial burden of chronic type 2 DKD is substantial [10], and prompt identification of chronic type 2 DKD along with proactive risk factor management might alleviate this financial strain [11]. Increased focus and effort are essential to tackle chronic type 2 DKD, which is an escalating public health issue impacting individuals globally [9]. This study aims to delineate the trends in the global, regional, and national prevalence of chronic type 2 DKD from 1990 to 2021, evaluate the impact of aging, population growth, and epidemiological change on the prevalence of chronic type 2 DKD, analyze inequalities among countries, and project changes up to 2040, thereby elucidating the significance of the disease prevalence attributable to chronic type 2 DKD.

## 2 Methods

### 2.1 Study Data

Utilizing recent epidemiological data and enhanced standardized methodologies, GBD 2021 (https://vizhub.healthdata.org/gbd‐results/) quantifies health loss for 371 diseases across 204 countries and territories, encompassing metrics of prevalence, disease severity, and mortality, which collectively provide a thorough evaluation of disease burden [12]. Numerous epidemiological studies have been conducted on a variety of diabetes types and their complications, utilizing the GBD database. These studies have offered recommendations for countries to allocate medical resources and develop public health policies [8, 13, 14]. This study gathered case numbers and age‐standardized rates (ASR) of chronic type 2 DKD categorized by sex, region, and country, spanning from 1990 to 2021. Simultaneously, the estimates and their 95% uncertainty interval (UI) for prevalence were obtained. The sociodemographic progress of a country or region was measured by the sociodemographic index (SDI), which represents the aggregate average of income, educational attainment, and fertility rates [15, 16]. The 204 countries and regions were subsequently categorized into five regions according to the SDI: low, low‐middle, middle, high‐middle, and high.

### 2.2 Data Analysis

ASR and estimated annual percentage change (EAPC) were employed to evaluate trends in the prevalence of chronic type 2 DKD. The EAPC, derived from a regression model that fits the natural logarithm to the calendar year, was computed to characterize the long‐term trend of ASR in the prevalence of chronic type 2 DKD, represented by the equation *Y* = *α* + *βX* + *ε*, where *y* = ln (rate), *x* = calendar year, and *ε* = error term. The EAPC is computed using the formula EAPC = 100 × (exp (*β*)−1). The 95% confidence intervals (CI) for the EAPC were derived from the linear model [8, 17]. If both the value of EAPC and the lower boundary of the 95% CI exceed 0, the age‐standardized indicator is deemed to exhibit an increasing trend. Conversely, if the EAPC value and the upper boundary of the 95% CI are both below 0, a decreasing trend is identified. A constant trend is indicated when the 95% CI of the EAPC encompasses 0 [17, 18]. We analyzed the raw case numbers by age, population, and epidemiological change, including age and population standardized prevalence, to assess the impact of population expansion, aging, and epidemiological change on chronic type 2 DKD during the past 30 years.

### 2.3 Cross‐Country Inequalities Analysis

The slope index of inequality and concentration index were employed to measure the SDI‐related disparity in the burden of chronic type 2 DKD among countries [19]. The slope index of inequality was determined by regressing the country‐level prevalence of chronic type 2 DKD across all age groups against the sociodemographic development‐related relative position scale, which is defined by the midpoint of the cumulative class range of the population ranked by SDI. The health inequality concentration index was determined by applying a Lorenz concentration curve to the observed cumulative relative distribution of populations ranked by SDI and disease prevalence, together with numerically integrating the region beneath the curve [20, 21].

### 2.4 Predictive Analysis

To predict the quantity and occurrence of new cases from 2020 to 2040, we employed a linear log age‐time‐cohort model that adeptly manages exponential growth trends and limits forecasts of linear trends. In conjunction with the estimating methodology and the resultant parameter estimates, the model was established utilizing the NORDPRED package in R program [22, 23]. The anticipated illness prevalence for 2040, for which data is accessible, is averaged and subsequently applied to the United Nations' population forecasts for each country for that year, enabling the forecasting of case numbers and the ASR to 2040 [22].

## 3 Results

### 3.1 Trend Analysis

**FIGURE 1:**
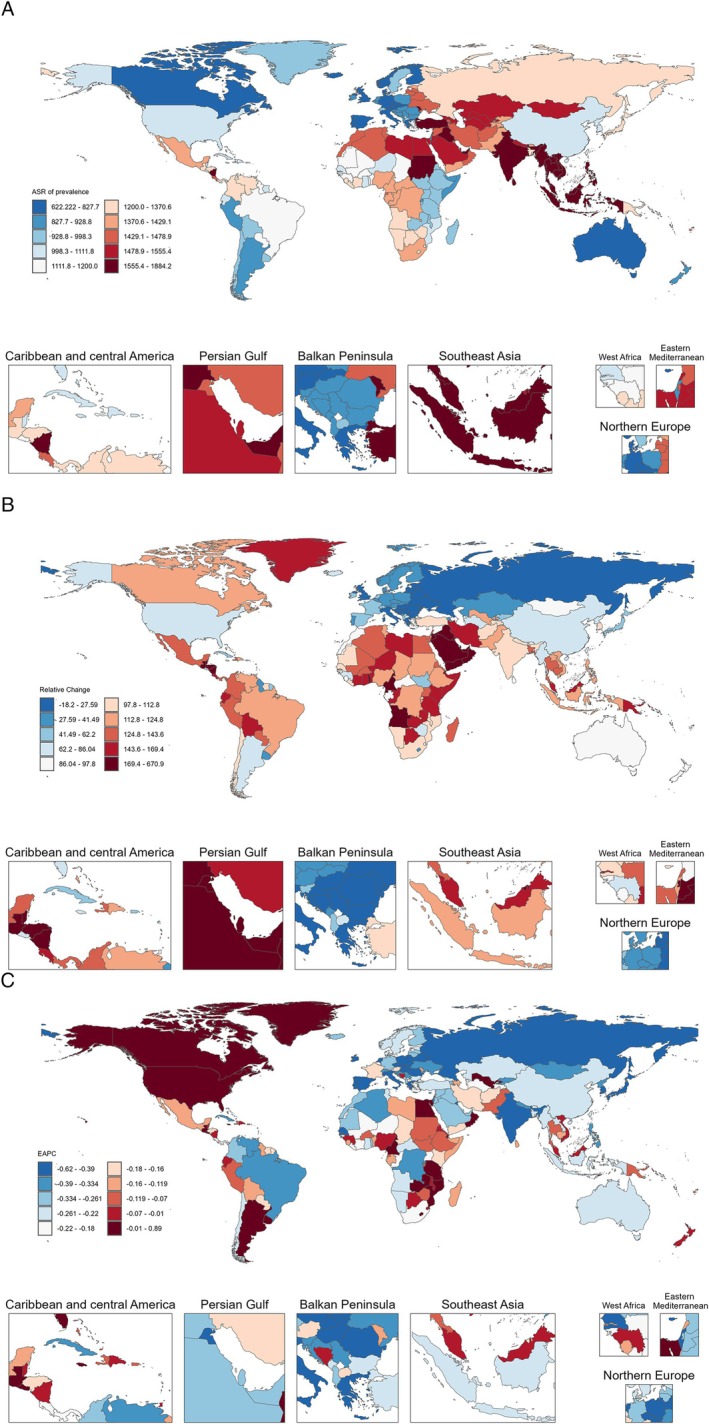
(A) The ASR of prevalence of chronic type 2 DKD in 2021. (B) The relative change in case number of prevalence of chronic type 2 DKD from 1990 to 2021. (C) The EAPC of ASR of prevalence of chronic type 2 DKD from 1990 to 2021. ASR, age‐standardized rate; EAPC, estimated annual percentage change.

Worldwide, the case number of chronic type 2 DKD rose from 58 105 268 (95% UI 53 056 992, 63 286 818) in 1990 to 107 559 955 (95% UI 99 170 797, 115 994 732) in 2021, reflecting a rise of 85.11%. In addition, the ASR for chronic type 2 DKD was 1327.22 (95% UI 1223.26, 1439.42) in 1990, 1259.63 (95% UI 1161.99, 1359.92) in 2021, and −0.17% (95% CI −0.20, −0.13) in EAPC, indicating stability. However, the majority of ASRs are experiencing a modest decline. In 1990 and 2021, the number of chronic type 2 DKD cases was slightly higher in females. However, the estimated annual percentage change (EAPC) in females was −0.14% (95% CI −0.19, −0.09), which was relatively smaller in magnitude compared to that in men (−0.21%, 95% CI −0.23, −0.18). The ASR exhibits significant variation across SDI regions, with the highest ASR shown in the low‐middle SDI region, at 1586.44 in 1990 (95% UI 1452.35, 1722.23) and 1474.59 in 2021 (95% UI 1357.94, 1606.52). The lowest ASR was 1037.34 (95% UI 959.27, 1110.52) in 1990 in the high SDI region and 997.07 (95% UI 918.7, 1066.28) in 2021 in the high SDI region. ASR diminished throughout all SDI regions. Moreover, the highest EAPC was elevated in the high SDI zone at −0.09% (95% CI −0.14, −0.05), whereas the low‐middle SDI region exhibited the lowest EAPC at −0.31% (95% CI −0.36, −0.27) (Table 1). In terms of 21 regions worldwide, in 2021, Southeast Asia had the highest ASR at 1739.34 (95% UI 1595.66, 1883.88), while Western Europe had the lowest ASR at 737.42 (95% UI 683.02, 790.09). In 1990, Southeast Asia had the highest ASR at 1793.6 (95% UI 1626.27, 1951.45), while Western Europe had the lowest ASR at 826.01 (95% UI 762.09, 883.39). From 1990 to 2021, the high‐income Asia Pacific region exhibited the lowest EAPC at −0.47% (95% CI −0.54, −0.41), while Eastern Sub‐Saharan Africa exhibited the highest EAPC at −0.05% (95% CI −0.07, −0.03). East Asia experienced minimal growth from 1990 to 2021, registering a decrease of 13.13%, with an EAPC of −0.25% (95% CI −0.39, −0.10). Nonetheless, high‐income North America experienced the most significant rise from 1990 to 2021 (4.94%), with an EAPC of 0.35% (95% CI 0.29, 0.42) (Figure 1A). The prevalence of chronic type 2 DKD was higher in most countries and regions in 2021 than in 1990 (Figure 1B). However, in most regions of the world, EAPC was less than 0, signifying a negative growth trend in ASR (Figure 1C).

**TABLE 1:** The case number and ASR of prevalence of chronic type 2 DKD in 1990 and 2021, and its temporal trends from 1990 to 2021.

Location	1990		2021		1990–2021
	Case number (95% UI)	ASR (95% UI)	Case number (95% UI)	ASR (95% UI)	EAPC (95% CI)
Global	58 105 268 (53 056 992, 63 286 818)	1327.22 (1223.26, 1439.42)	107 559 955 (99 170 797, 115 994 732)	1259.63 (1161.99, 1359.92)	−0.17% (−0.20, −0.13)
Sex					
Male	29 744 708 (27 057 208, 32 317 657)	1414.09 (1298.44, 1528.78)	54 722 423 (50 395 175, 59 068 229)	1333.1 (1229.35, 1437.39)	−0.21% (−0.23, −0.18)
Female	28 360 560 (25 996 835, 30 841 479)	1250.08 (1152.38, 1357.8)	52 837 532 (48 769 234, 56 931 920)	1191.96 (1100.99, 1286.63)	−0.14% (−0.19, −0.09)
SDI					
High SDI	11 068 895 (10 251 403, 11 847 690)	1037.34 (959.27, 1110.52)	18 097 946 (16 763 917, 19 359 376)	997.07 (918.7, 1066.28)	−0.09% (−0.14, −0.05)
High‐middle SDI	13 233 847 (12 089 212, 14 479 468)	1271.32 (1167.77, 1384.68)	21 060 241 (19 314 069, 22 837 225)	1157 (1062.14, 1253.04)	−0.25% (−0.32, −0.19)
Middle SDI	17 975 998 (16 224 751, 19 689 647)	1431.89 (1315.46, 1554.26)	36 412 911 (33 598 848, 39 348 243)	1331.6 (1232.11, 1433.53)	−0.28% (−0.25, −0.16)
Low‐middle SDI	11 948 110 (10 785 488, 13 071 618)	1586.44 (1452.35, 1722.23)	23 752 428 (21 773 065, 25 995 904)	1474.59 (1357.94, 1606.52)	−0.31% (−0.36, −0.27)
Low SDI	3 827 766 (3 462 237, 4 236 705)	1355.23 (1238.78, 1484.31)	8 155 174 (7 427 183, 9 026 803)	1269.37 (1167.02, 1378.81)	−0.28% (−0.32, −0.25)
Region					
Andean Latin America	244 091 (215 794, 276 279)	983.26 (882.52, 1092.28)	597 139 (538 461, 662 179)	957.16 (862.93, 1056.15)	−0.06% (−0.10, −0.03)
Australasia	193 267 (174 191, 211 086)	827.63 (744.93, 908.08)	378 015 (344 562, 414 094)	768.63 (694.98, 843.15)	−0.20% (−0.27, −0.13)
Caribbean	318 976 (288 671, 353 374)	1115.04 (1013.04, 1223.76)	563 203 (515 384, 614 522)	1069.87 (978.55, 1169.72)	−0.15% (−0.17, −0.13)
Central Asia	806 014 (735 985, 881 356)	1563.79 (1441.41, 1698.32)	1 315 604 (1 205 400, 1 431 741)	1493.98 (1381.44, 1617.53)	−0.16% (−0.18, −0.15)
Central Europe	1 359 625 (1 251 502, 1 475 288)	938.65 (865.79, 1016.66)	1 685 180 (1 564 183, 1 814 449)	855.75 (792.62, 922.18)	−0.38% (−0.41, −0.36)
Central Latin America	1 429 952 (1 296 461, 1 574 606)	1408.19 (1294.38, 1525.56)	3 407 649 (3 135 740, 3 667 865)	1327.57 (1225.04, 1427.15)	−0.18% (−0.20, −0.15)
Central Sub‐Saharan Africa	421 277 (371 461, 475 086)	1480.03 (1343.41, 1620.91)	976 815 (874 266, 1 085 754)	1377.49 (1262.91, 1506.7)	−0.32% (−0.34, −0.29)
East Asia	12 310 493 (11 172 806, 13 525 325)	1213.4 (1107.78, 1317.9)	21 662 039 (19 882 230, 23 414 195)	1054.1 (972.27, 1140.11)	−0.25% (−0.39, −0.10)
Eastern Europe	4 067 469 (3 722 843, 4 450 527)	1537.86 (1409.57, 1677.58)	4 315 524 (3 951 875, 4 696 855)	1390.26 (1277.42, 1520.38)	−0.38% (−0.40, −0.36)
Eastern Sub‐Saharan Africa	942 947 (844 299, 1 045 473)	952.85 (866.99, 1041.33)	2 183 334 (1 987 628, 2 407 174)	942.79 (861.6, 1032.88)	−0.05% (−0.07, −0.03)
High‐income Asia Pacific	2 905 294 (2 658 369, 3 157 585)	1446.13 (1324.25, 1571.58)	4 607 841 (424 357 8, 494 698 2)	1275.44 (1168.23, 1377.36)	−0.47% (−0.54, −0.41)
High‐income North America	3 432 141 (3 185 380, 3 678 787)	1006.79 (934.2, 1082.15)	6 203 134 (5 758 563, 6 652 332)	1056.52 (979.59, 1135.34)	0.35% (0.29, 0.42)
North Africa and Middle East	3 300 457 (2 973 590, 3 643 974)	1569.05 (1421.63, 1723.9)	8 039 618 (7 267 276, 8 805 018)	1505.85 (1368.99, 1642.89)	−0.15% (−0.17, −0.14)
Oceania	55 403 (47 748, 61 986)	1396.98 (1240.14, 1537.7)	127 252 (1 11 037, 142 277)	1337.24 (1193.94, 1478.48)	−0.15% (−0.16, −0.13)
South Asia	12 503 454 (11 237 789, 13 777 983)	1712.63 (1562.29, 1863.11)	25 462 949 (23 248 464, 27 987 294)	1547.31 (1418.44, 1687.6)	−0.44% (−0.52, −0.36)
Southeast Asia	5 753 417 (5 149 626, 6 345 741)	1793.6 (1626.27, 1951.45)	12 333 536 (11 263 092, 13 439 669)	1739.34 (1595.66, 1883.88)	−0.18% (−0.23, −0.13)
Southern Latin America	413 284 (371 175, 461 923)	885.38 (796.36, 987.75)	753 905 (680 383, 837 548)	911.84 (819.79, 1021.13)	0.13% (0.11, 0.15)
Southern Sub‐Saharan Africa	461 703 (421 236, 508 339)	1418.23 (1302.09, 1533.13)	890 371 (815 494, 968 329)	1362.72 (1254.88, 1470.95)	−0.17% (−0.19, −0.16)
Tropical Latin America	1 356 933 (1 224 045, 1 486 784)	1266.4 (1153.22, 1371.28)	2 960 767 (2 714 465, 3 207 229)	1145.84 (1053.26, 1238.67)	−0.36% (−0.38, −0.35)
Western Europe	4 413 774 (4 054 376, 4 720 538)	826.01 (762.09, 883.39)	5 879 101 (5 438 217, 6 289 341)	737.42 (683.02, 790.09)	−0.41% (−0.47, −0.35)
Western Sub‐Saharan Africa	1 415 298 (1 293 966, 1 541 607)	1328.43 (1227.41, 1428.43)	3 216 978 (2 963 598, 3 492 631)	1276.31 (1183.6, 1373.75)	−0.11% (−0.12, −0.10)

### 3.2 Decomposition Analysis

**FIGURE 2:**
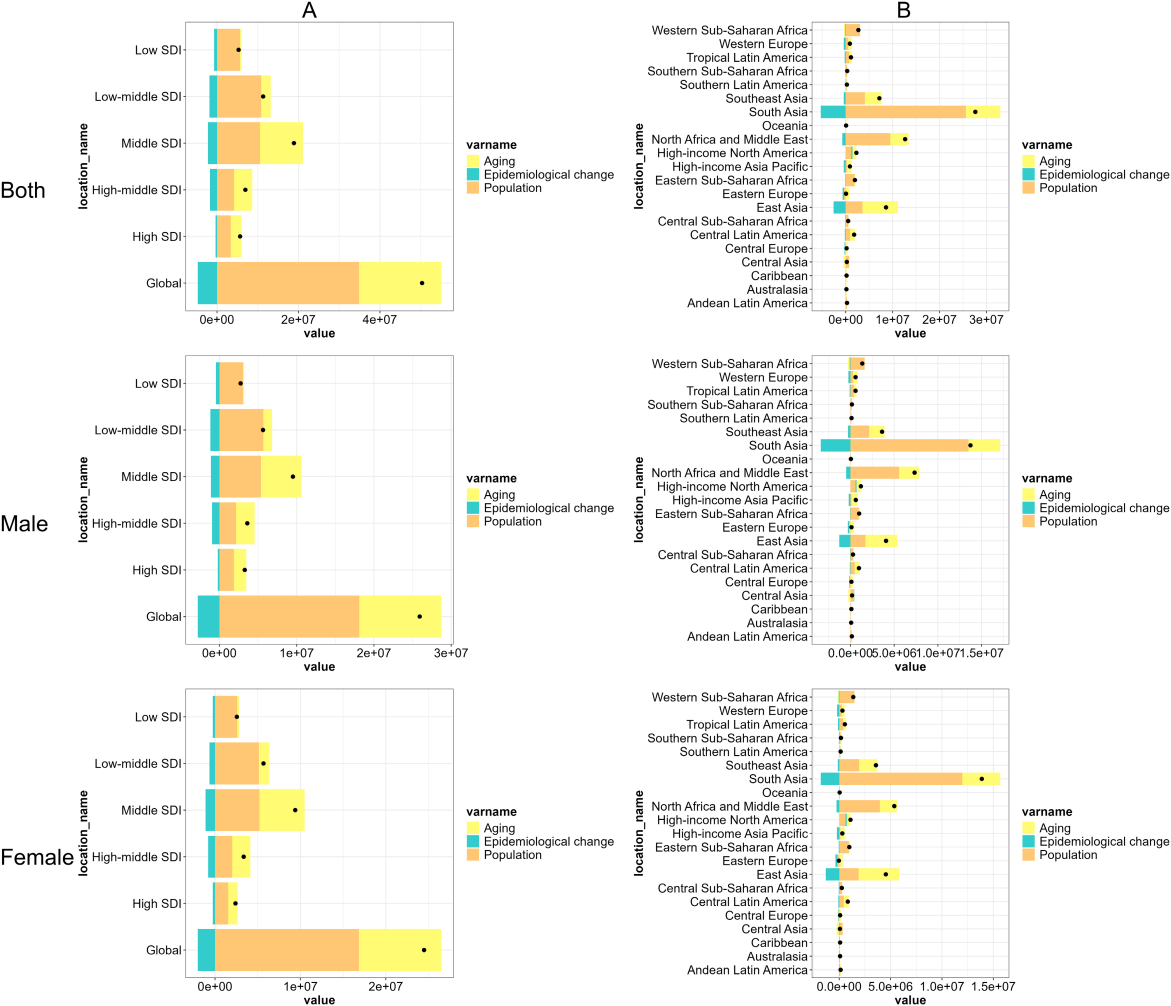
Changes in prevalence of chronic type 2 DKD according to population‐level determinants including aging, population growth, and epidemiological change from 1990 to 2021 at the global level and by SDI quintiles (A) and regions (B) stratified by sexes. SDI, sociodemographic index.

Globally, from 1990 to 2021, population growth has been the dominant driver of the disease burden in the prevalence of chronic type 2 DKD, followed by aging, while epidemiological change has mitigated this burden. This pattern was consistent across the five SDI regions, though subtle variations were observed among the 21 GBD regions. Importantly, no gender‐specific differences were noted in this overarching trend (Figure 2).

### 3.3 Cross‐Country Inequality Analysis

**FIGURE 3:**
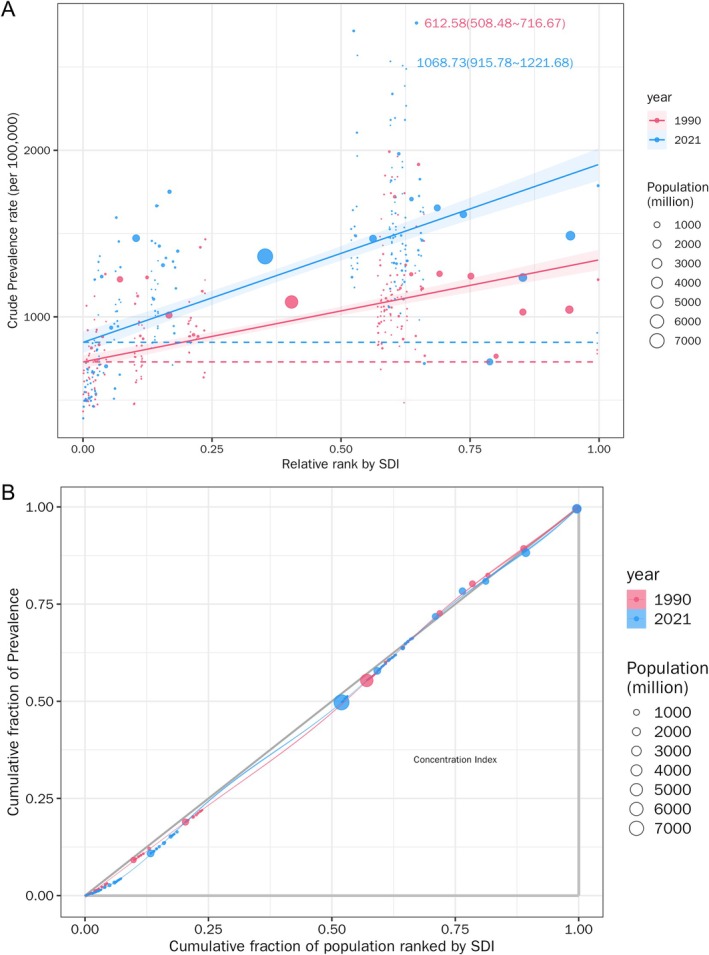
Health inequality regression curves (A) and concentration curves (B) for the prevalence of chronic type 2 DKD from 1990 to 2021 across the world.

Significant absolute and relative SDI‐related inequalities in the burden of chronic type 2 DKD were observed, with notable increases in these indicators with time (Figure 3). A greater prevalence was seen to disproportionately cluster in countries with elevated SDI. In 1990, the slope index of inequality was 534.02, indicating a prevalence excess of 534.02 (per 100 000 population) in the country with the highest SDI compared to the country with the lowest SDI. This inequality increased to 930.71 by 2021. Simultaneously, the concentration index exhibited an upward trend from 1990 to 2021.

### 3.4 Predicted Trends

**FIGURE 4:**
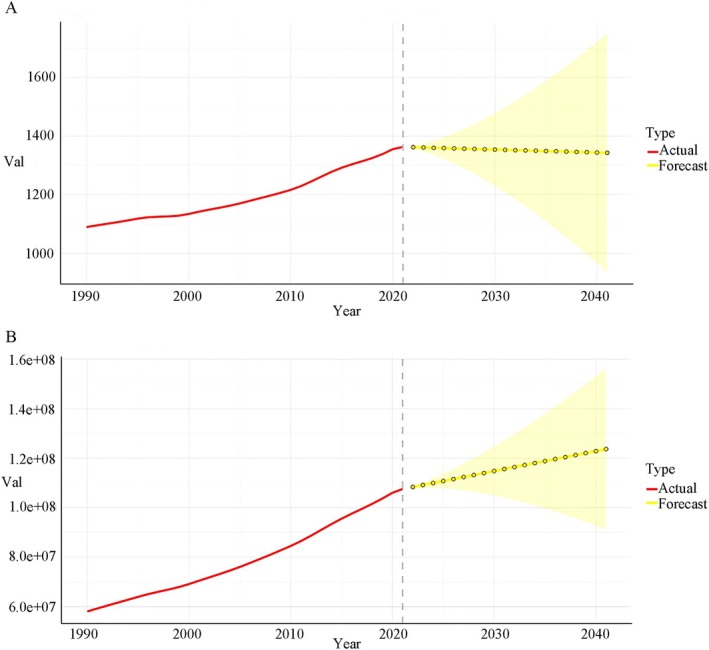
(A) The global change trends of ASR of prevalence of chronic type 2 DKD from 1990 to 2021, and its predicted trends between 2022 and 2042. (B) The global change trends of case number of prevalence of chronic type 2 DKD from 1990 to 2021, and its predicted trends between 2022 and 2042.

The prevalence of chronic type 2 DKD escalates with age and population dynamics, influenced by various factors. We projected prevalence case numbers and ASR for chronic type 2 DKD are outlined for the years 2020–2040 at global levels. Globally, the total number of cases will markedly rise over the next 20 years, while the prevalence rate will remain relatively stable (Figure 4A,B).

## 4 Discussion

The global pandemic of type 2 diabetes represents a significant health burden, with DKD emerging as the primary cause of end‐stage renal disease, a prevalent complication of diabetes worldwide [24]. Chronic DKD is the most perilous consequence of diabetes, contributing to increased morbidity and death in individuals with type 1 and type 2 diabetes [25]. Given that type 2 diabetes is a significant contributor to chronic DKD, it is essential to prioritize research in this area. Currently, there is no research focused solely on chronic type 2 DKD. This study is the inaugural investigation into the global prevalence of this disease, as per the GBD database.

In 2021, the global prevalence of chronic type 2 DKD exceeded 107 million cases, predominantly occurring in regions with middle and low‐middle SDI regions. Our data indicated that these regions had elevated ASR and diminished EAPC relative to high SDI regions, likely attributable to a greater ASR baseline in middle SDI and low‐middle SDI countries compared to 1990. Nonetheless, all SDI countries have exhibited a marginal decrease in ASR as the prevalence of chronic type 2 DKD has gained greater acknowledgment in recent years, health conditions have improved, and the prevention and treatment of chronic type 2 DKD have intensified [8]. The World Health Organization (WHO) Global Diabetes Compact was founded in 2021 to enhance healthcare access for individuals with diabetes and to collaborate closely with them. The United Nations employs diabetes treatment as a metric for evaluating the healthcare systems of nations in relation to universal health coverage goals [26] and has set a target to decrease premature mortality from diabetes and other non‐communicable diseases by one‐third by 2030, as outlined in Goal 3 of the United Nations Sustainable Development Goals [27]. Consequently, these alterations may be associated with the initiatives undertaken by the WHO and the United Nations.

Population growth and aging have significantly contributed to the rising prevalence of chronic type 2 DKD globally, regionally, and nationally; however, epidemiological change have mitigated this trend. Therefore, priorities should be placed on addressing the disease burden caused by population growth—such as expanding access to medical resources and strengthening public health planning. Although aging contributes less to the DKD burden than population growth, as the second most important driver, it still needs to be incorporated into long‐term public health planning. Meanwhile, efforts should continue to prioritize the promotion of epidemiological interventions (e.g., diabetes management, lifestyle modifications), especially with precision adjustments tailored to GBD regions showing significant regional variations [8]. The consistency across genders indicates that there is no need to design gender‐specific prevention and control strategies, allowing resources to focus on universal interventions.

An in‐depth investigation of health inequalities reveals that the prevalence of chronic type 2 DKD is significantly concentrated in impoverished and underdeveloped regions characterized by inadequate screening capabilities and limited therapeutic interventions. Income is fundamental to population health, and its disparity significantly influences health inequality since higher income typically correlates with increased access to medical treatment and improved health outcomes [28]. High SDI countries possess more modern medical facilities, more comprehensive healthcare measures, and greater reserves of medical talent compared to low to medium SDI countries [8]. For instance, not all patients with DKD are eligible for renal replacement therapy, and 78% of these patients reside in low‐ and middle‐income countries, where resources, dialysis availability, and kidney donations are constrained [29]. In sub‐Saharan Africa, although individuals in need of renal replacement therapy commence treatment, retention rates are low due to an inability to afford continuous dialysis, resulting in up to 85% of new patients refusing this life‐saving intervention [30]. Public health policy contributes to reducing the prevalence rate of DKD by educating healthcare professionals, establishing early kidney disease detection programs, implementing nephroprotective treatments, and appropriately managing CKD risk factors such as elevated systolic blood pressure and glucose levels [31]. This measure may be particularly significant in regions with low SDI, where primary care health services prioritize infant and maternal health and are less capable of effectively preventing and managing chronic diseases [32]. Furthermore, fewer than 50% of the patients underwent testing for urine albumin, an initial indicator of renal damage induced by diabetes [33]. Numerous nations continue to be deficient in a proficient cadre of nephrology specialists and universal access to primary healthcare and renal replacement medication. Screening for renal function in diabetes patients, along with increasing awareness, is essential for the early identification of CKD. The alleviation of chronic type 2 DKD should be incorporated into governmental health goals and resource distribution strategies, emphasizing prevention, early management, and the postponement of disease progression.

Forecasts derived from current data indicate that the prevalence of chronic type 2 DKD has not diminished, and the emergence of new cases will remain significant in most nations due to population expansion and ongoing aging trends. The majority of countries will exhibit a significant prevalence of chronic type 2 DKD. Notwithstanding advancements in screening for chronic type 2 DKD by 2040, the prevalence of chronic type 2 DKD is projected to rise gradually from 2021 to 2040, owing to its incurable nature and the significant correlation between aging and chronic type 2 DKD. The alleviation of diabetes mellitus–related CKD should be incorporated into the government's health priorities and budget allocation strategies, emphasizing prevention, early management, and the postponement of disease progression.

This study possesses certain limitations: (1) The GBD study mostly relies on collected data; however, the data collection methods, techniques, and tools vary across countries, resulting in potentially inconsistent cross‐country evidence, making it difficult to distinguish between high‐quality and low‐quality data. (2) Diagnostic rates may fluctuate based on the degree of development and healthcare quality in different countries, potentially leading to underdiagnosis in nations with inferior healthcare systems. Analyzing the temporal trends in the prevalence of chronic type 2 DKD across 204 countries and territories over the past 30 years, along with forecasting future changes over the next 20 years, can enhance the understanding of disease epidemiology in various nations, potentially guiding the formulation of tailored health policies to address specific disease burdens globally. Conversely, examining the cross‐national disparities in disease burden may enhance our comprehension of the SDI‐related distributional inequalities for chronic type 2 DKD, thereby facilitating a judicious allocation of scarce medical resources globally, which could promote global health.

## 5 Conclusion

In conclusion, chronic type 2 DKD is a major public health concern of worldwide significance. The rising prevalence of chronic type 2 DKD attributable to population growth and epidemiological change poses a significant challenge for developing nations; conversely, the issue of chronic type 2 DKD resulting from modernization and urbanization is expected to gain prominence in developed countries in the near future. Moreover, although the prevalence of chronic type 2 DKD is skewed towards less‐developed countries, this burden is disproportionately more significant in places with elevated sociodemographic development and is escalating over time. Implementing healthy lifestyle habits and implementing tailored tactics based on varying socioeconomic conditions in countries have been suggested as feasible and successful management approaches. The widespread occurrence of chronic type 2 DKD surpasses current public awareness, necessitating further research to explore its determinants and address potential causes, ultimately aiding in the prevention of chronic type 2 DKD and reducing the global disease burden.

## Author Contributions

**Yujun He:** writing – original draft, writing – review and editing, conceptualization, methodology, formal analysis, data curation, supervision, project administration. **Xiaoyi Wang:** writing – review and editing, methodology, investigation, data curation, visualization. **Lu Li:** writing – review and editing, conceptualization, investigation. **Minhui Liu:** writing – review and editing, conceptualization, methodology, formal analysis, data curation, visualization. **Ru Chen:** writing – review and editing, investigation, data curation, validation. **Yachao Wu:** writing – review and editing, formal analysis. **Jiujie He:** writing – review and editing, investigation, data curation. **Wei Mai:** writing – review and editing, investigation. **Xiaojun Li:** writing – review and editing, project administration, funding acquisition.

## Ethics Statement

The authors have nothing to report.

## Consent

The authors have nothing to report.

## Conflicts of Interest

The authors declare no conflicts of interest.

## Data Availability

The datasets utilized in this investigation are accessible in open repositories. The repository names and accession numbers are provided below: All data may be accessed via the IHME website (https://vizhub.healthdata.org/gbd‐results/).
